# Quantitative Proteomics Analysis Reveals the Function of the Putative Ester Cyclase UvEC1 in the Pathogenicity of the Rice False Smut Fungus *Ustilaginoidea virens*

**DOI:** 10.3390/ijms22084069

**Published:** 2021-04-15

**Authors:** Xiaoyang Chen, Zhangxin Pei, Pingping Li, Xiabing Li, Yuhang Duan, Hao Liu, Xiaolin Chen, Lu Zheng, Chaoxi Luo, Junbin Huang

**Affiliations:** The Key Lab of Plant Pathology of Hubei Province, Huazhong Agricultural University, Wuhan 430070, China; chenxiaoyang2345@163.com (X.C.); peizhangxinhzau@163.com (Z.P.); LPP_19960202@163.com (P.L.); lixiabinghzau@163.com (X.L.); duan199607272021@163.com (Y.D.); hl210@mail.hzau.edu.cn (H.L.); chenxiaolin@mail.hzau.edu.cn (X.C.); luzheng@mail.hzau.edu.cn (L.Z.); cxluo@mail.hzau.edu.cn (C.L.)

**Keywords:** *Ustilaginoidea virens*, ester cyclase, virulence, proteomic

## Abstract

Rice false smut is a fungal disease distributed worldwide and caused by *Ustilaginoidea virens*. In this study, we identified a putative ester cyclase (named as UvEC1) as being significantly upregulated during *U. virens* infection. UvEC1 contained a SnoaL-like polyketide cyclase domain, but the functions of ketone cyclases such as SnoaL in plant fungal pathogens remain unclear. Deletion of *UvEC1* caused defects in vegetative growth and conidiation. *UvEC1* was also required for response to hyperosmotic and oxidative stresses and for maintenance of cell wall integrity. Importantly, Δ*UvEC1* mutants exhibited reduced virulence. We performed a tandem mass tag (TMT)-based quantitative proteomic analysis to identify differentially accumulating proteins (DAPs) between the Δ*UvEC1-1* mutant and the wild-type isolate HWD-2. Proteomics data revealed that UvEC1 has a variety of effects on metabolism, protein localization, catalytic activity, binding, toxin biosynthesis and the spliceosome. Taken together, our findings suggest that *UvEC1* is critical for the development and virulence of *U. virens*.

## 1. Introduction

Rice false smut (RFS), caused by the pathogenic ascomycete fungus *Ustilaginoidea virens* (*U. virens*), is one of the most devastating grain diseases in the majority of rice-growing areas of the world [1]. Conidia germinate on the surface of rice (*Oryza sativa*) spikelets, and the emerging hyphae extend randomly and enter the inner space of spikelets through small gaps between lemma and palea to colonize floral organs [2,3]. Once inside these organs, the pathogen first attacks the filaments, thus preventing pollen from maturing and blocking normal fertilization, and then invades the stigma and style to simulate fertilization, resulting in the hijacking of nutrients normally earmarked for developing seeds to form rice false smut balls [4,5]. The occurrence of this disease not only causes yield loss, but also threatens human and animal health by producing cyclopeptide mycotoxins, including ustilaginoidins, ustiloxins and sorbicillinoids [6,7,8,9,10]. Genomic and transcriptomic analyses of *U. virens* infection of rice have shown that this fungus produces many pathogenicity factors [11]. However, a limited molecular determinant of the virulence of *U. virens* have been experimentally verified [12]. RFS is difficult to control due to that no resistant genes or completely immune rice varieties have been identified. Therefore, understanding of the pathogenic mechanism of *U. virens* to provide more effective strategies to control RFS.

Polyketides (PKs) are a large, diverse group of natural compounds produced mainly by bacteria, fungi and plants, with high structural diversity and multiple biological activities [13,14]. Many of these compounds and their derivatives have been used in the field of medicine, including lovastatin, tetracycline and erythromycin [15,16,17]. However, many polyketides pose serious threats to the food industry, agriculture and human health due to their acute toxicity, such as the mycotoxin T-toxin, fumonisin and aflatoxins [18,19,20,21,22]. The deletion of the genes encoding polyketide synthase (UvPKS1), laccase (UgsL), the major facilitator superfamily (MFS) transporter UgsT and the FAD binding domain protein UgsO lead to a reduction in ustilaginoidin-related products, indicating that these genes are involved in ustilaginoidin biosynthesis [23]. Fungal polyketide synthases are key enzymes in the biosynthesis of fungal polyketides and belong to the class of multifunctional enzymes, as they also harbor functional domains with cyclase, thioesterase and methyltransferase activities. In regard to catalytic mechanism, most fungal polyketide synthases catalyzes the biosynthesis of polyketides with acetyl CoA as the starting substrate, malonyl CoA as the extension unit and repeated Claisen condensation to extend the polyketide chain [14]. SnoaL is a polyketide cyclase that adopts a distorted alpha-beta barrel fold, the polyketide cyclases thus form of family of enzymes with a unique catalytic strategy for aldol condensation [24]. For example, SnoaL belongs to a family of small polyketide cyclases that catalyze ring closure steps during the biosynthesis of polyketide antibiotics in *Streptomyces*. While SnoaL-like polyketide cyclases are clearly involved in the biosynthesis of polyketide antibiotics, to date only a few proteins with this functional domain have been reported, with a focus on their biochemical and chemical functions. AknH from *Streptomyces galilaeus*, for instance, is a small polyketide cyclase that catalyzes both the closure of the fourth carbon ring during the biosynthesis of aclarubicin and the conversion of aclarubicin acid methyl ester to aclarubicin ketone [25]. Aklanonic acid methyl ester cyclase is involved in the synthesis of aklavinone, an intermediate in the biosynthetic pathway of clinically important anthracyclines such as daunorubicin and doxorubicin [26]. Comparative genome analyses evidenced the presence of a SnoaL cyclase, its putative role in the polyketide cyclization reaction of the ochratoxin A (OTA) biosynthesis pathway of *Aspergillus* and *Penicillium* [27]. However, the function of cyclases in fungal pathogens is still unknown.

Here, we report that the deletion of the putative ester cyclase gene *UvEC1* causes defects in mycelial growth, conidiation, stress response and virulence. To investigate the cellular and molecular bases of *UvEC1*-mediated virulence, we undertook a tandem mass tag (TMT)-based quantitative proteomic approach to discover differentially accumulating proteins between the wild-type *U. virens* isolate HWD-2 and a Δ*UvEC1-1* deletion mutant. This study provides novel insights into the role of the putative ester cyclase in fungal development and virulence.

## 2. Results

### 2.1. Identification and Characterization of UvEC1 in U. virens

**Figure 1:**
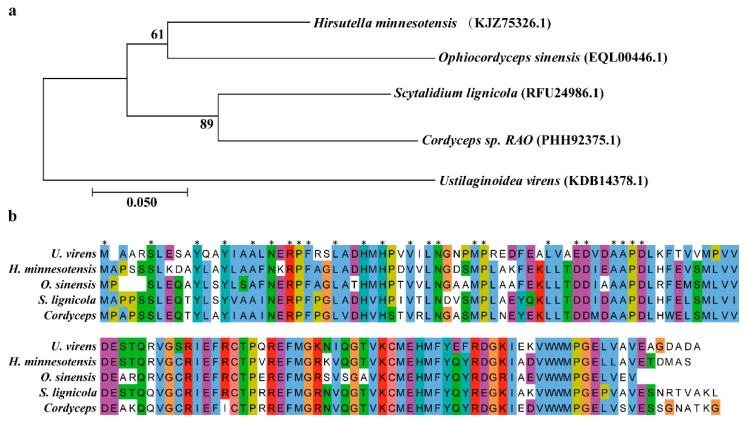
Characterization of UvEC1 in *U. virens*. (**a**) Neighbor-joining phylogenetic tree of putative EC1 homologs encoded by 5 fungal genomes generated with MEGA7.0. The numbers at each branch node are bootstrap percentages out of 1000 replicates. (**b**) The mutiple alignment of EC1 homologs proteins amino acid sequences of *H. minnesotensis*, *S. lignicola*, *Cordyceps* and *O. sinensis*.

In the RNA-seq data of *U. virens* infecting rice spikilets [28], we found that the Uv8b_4649 gene was significantly up-regulated during the infection process (Figure S1), we speculated that it may be involved in the pathogenic process of *U. virens*. Uv8b_4649 (GenBank ID KDB14378.1) function annotation as a putative ester cyclase, which consisted of a 402-bp open reading frame encoding a 133-amino-acid protein. The encoded protein harbored a polyketide cyclase SnoaL domain, located between amino acids 7–127. A basic local alignment search tool for protein (BLASTP) search for SnoaL domain homologs at the NCBI database identified proteins with high similarity in other fungi, including *Hirsutella minnesotensis* (69.84%), *Scytalidium lignicola* (66.4%)*,* *Cordyceps sp. RAO* (66.14%) and *Ophiocordyceps sinensis* (65.57%) (Figure 1a). Multiple amino acid sequence alignment showed that they had a conserved ketocyclase SnoaL domain (Figure 1b). Based on a BLASTP analysis, EC1 homologous proteins appeared to be specifically distributed in bacteria and a few fungi.

### 2.2. Genomic Deletion and Complementation of UvEC1 in U. virens

**Figure 2:**
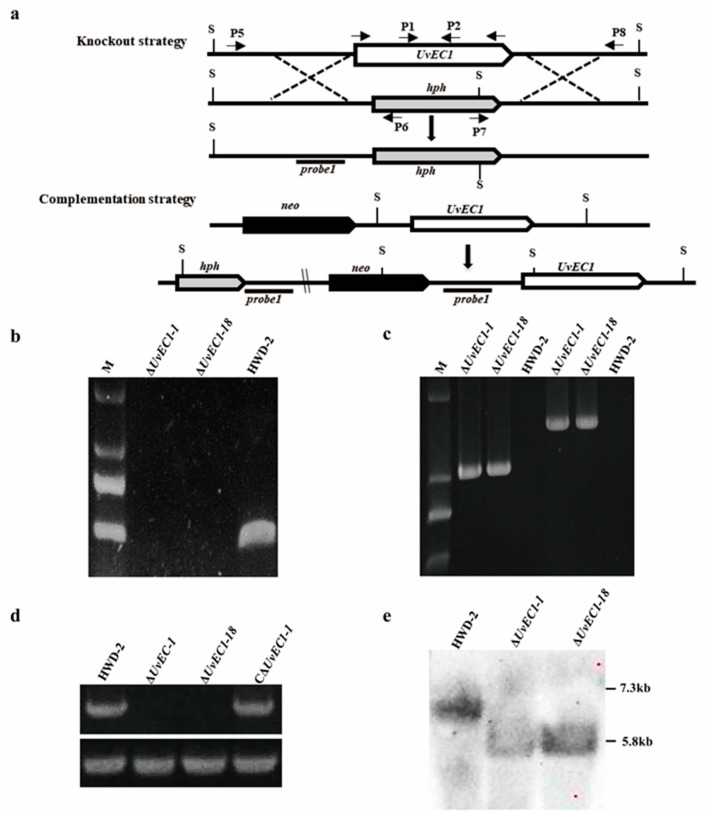
Targeted gene deletion and complementation of *UvEC1* in *U. virens*. (**a**) Knockout and complementation strategies for *UvEC1*. (**b**–**d**) RT-PCR analyses of *UvEC1* expression in *UvEC1* mutants and complementation strains. (**e**) Southern blot analysis of HWD-2 and Δ*UvEC1* mutants.

To investigate the biological functions of *UvEC1* in *U. virens*, we deleted the *UvEC1* gene by homologous recombination (Figure 2a). From our *UvEC1* knock-out library, we identified two independent deletion mutants with similar phenotypes by genomic PCR (Figure 2b,c) with the primers listed in Table S1. We validated the putative *UvEC1* knockout mutants (∆*UvEC1-1* and ∆*UvEC1-18*) by RT-PCR and Southern blot analyses (Figure 2d,e). Indeed, RT-PCR demonstrated that *UvEC1* was expressed only in the wild-type strain HWD-2 but not in either knockout mutant (Figure 2d). Similarly, Southern blot analysis revealed a single 5.8-kb band in the *UvEC1* knockout mutants that was shorter than that detected in HWD-2 (7.3 kb) (Figure 2e). To ascertain whether any of the phenotypes observed in *UvEC1* deletion mutants would be restored by the reintroduction of a wild-type copy of *UvEC1*, we also generated a complementation line in the ∆*UvEC1-1* mutant background. Accordingly, we introduced a *UvEC1*-*GFP* construct placed under the control of the native *UvEC1* promoter, by Agrobacterium (*Agrobacterium tumefaciens*)-mediated transformation. We confirmed the nature of the complementation strain C∆*UvEC1-1* by RT-PCR analysis for *UvEC1* transcript levels (Figure 2e). We thereby determined the phenotypes associated with the wild-type strain HWD-2, the deletion mutants ∆*UvEC1-1* and ∆*UvEC1-18* and the complementation strain C∆*UvEC1-1*.

### 2.3. Deletion of UvEC1 Affects Vegetative Growth and Conidiation of U. virens

**Figure 3:**
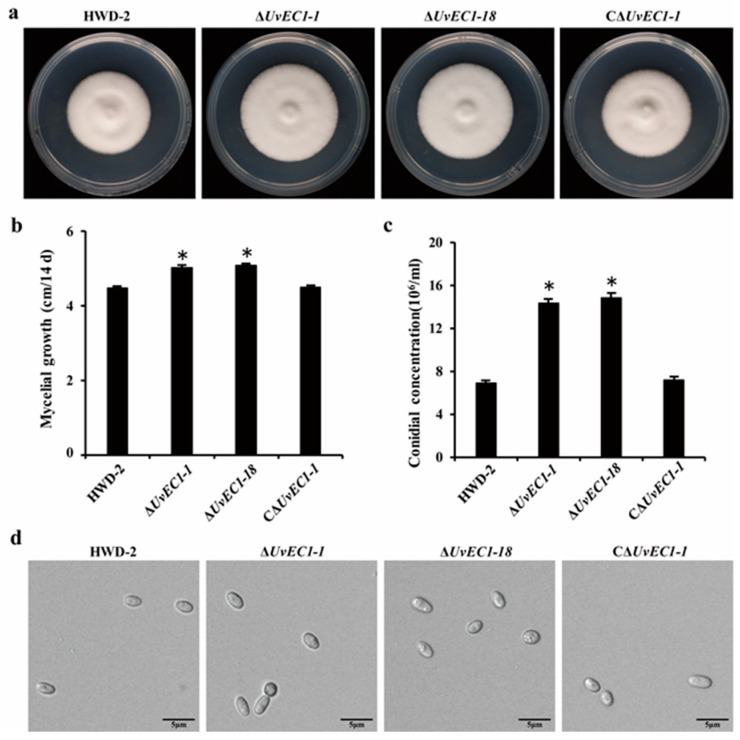
Deletion of *UvEC1* affects vegetative growth and conidiation of *U. virens*. (**a**) Colony morphology of the wild type HWD-2, ∆*UvEC1* mutants and the complementation strain on potato sucrose agar (PSA) medium after 14 days of growth in the dark at 28 °C. (**b**) Mean colony diameter of the strains shown in a. (**c**) Mean conidial production of the wild type HWD-2, the ∆*UvEC1* mutants and complementation strain grown in potato sucrose broth (PSB) medium at 28 °C with shaking at 180 rpm for 7 days. (**d**) Conidia morphology of the wild type HWD-2, the ∆*UvEC1* mutants and complementation strain. Scale bar = 5 μm. Asterisks represent significant differences relative to HWD-2, as determined by Least Significant Difference (LSD) at *p* = 0.05.

As compared to the wild-type strain HWD-2, the ∆*UvEC1* mutants exhibited a significantly higher mycelial growth rate (Figure 3a,b), although an examination of mycelial morphology under the microscope showed no apparent differences between ∆*UvEC1* mutants and HWD-2. Conidiation was significantly increased in ∆*UvEC1* mutants relative to the wild-type strain, but mutant conidia appeared morphologically normal (Figure 3c,d). These results indicated that *UvEC1* plays important roles in the vegetative growth and conidiation of *U. virens*.

### 2.4. UvEC1 Is Important for Response to Various Stresses

**Figure 4:**
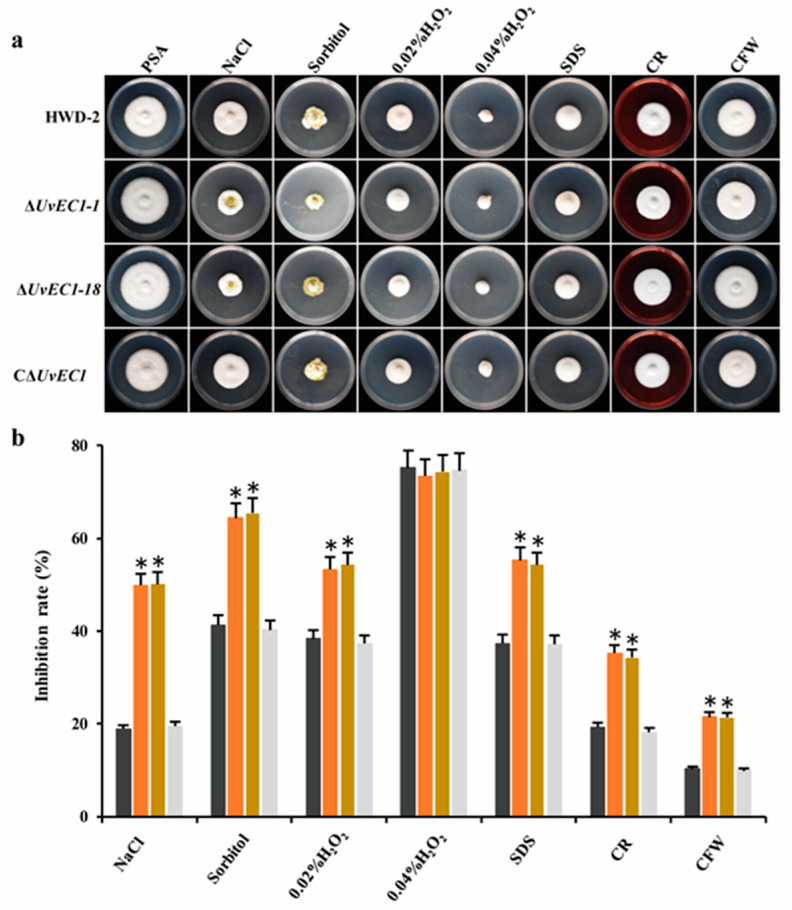
Sensitivity of ∆*UvEC1* mutants to various stresses. (**a**) Colony growth of HWD-2, *UvEC1* mutants and the complementation strain on PSA medium containing various stress inducers after 14 days at 28 °C in the dark. (**b**) Rates of growth inhibition of colonies by the stress inducers shown in a. Asterisks represent significant differences relative to HWD-2, as determined by LSD at *p* = 0.05.

To evaluate the role of *UvEC1* in mediating adaptation of *U. virens* to morphogenesis- or pathogenesis-associated stresses, we compared radial growth rates in WT, the ∆*UvEC1* mutants and the complementation strain grown on PSA medium containing various stress agents. Among all chemicals tested, 0.3 M NaCl and 0.5 M sorbitol significantly repressed mycelial growth of ∆*UvEC1* mutants compared to that of HWD-2. Likewise, stress agents affecting the cell wall, such as 0.03% SDS, 120 μg/mL Congo red (CR) or 120 μg/mL calcofluor white (CFW), significantly slowed the growth of the ∆*UvEC1* mutants relative to the wild-type strain HWD-2 (Figure 4a,b). By contrast, 0.04% H_2_O_2_ did not differentially inhibit mycelial growth, although all strains did show a strong growth limitation under this treatment. We hypothesized that the concentration used might have imposed a strong effect, resulting in no clear differential growth inhibition. We therefore repeated the experiment with a lower dose of 0.02% H_2_O_2_, which significantly slowed mycelial growth of the ∆*UvEC1* mutants relative to wild type HWD-2 (Figure 4a,b). Importantly, all growth defects observed in the ∆*UvEC1* deletion mutants were fully rescued in the C∆*UvEC1* complementation strain, indicating that *UvEC1* plays a key role in response to oxidative stress, osmotic stress and cell wall integrity in *U. virens.*

### 2.5. Deletion of UvEC1 Affects the Pathogenicity of U. virens

**Figure 5:**
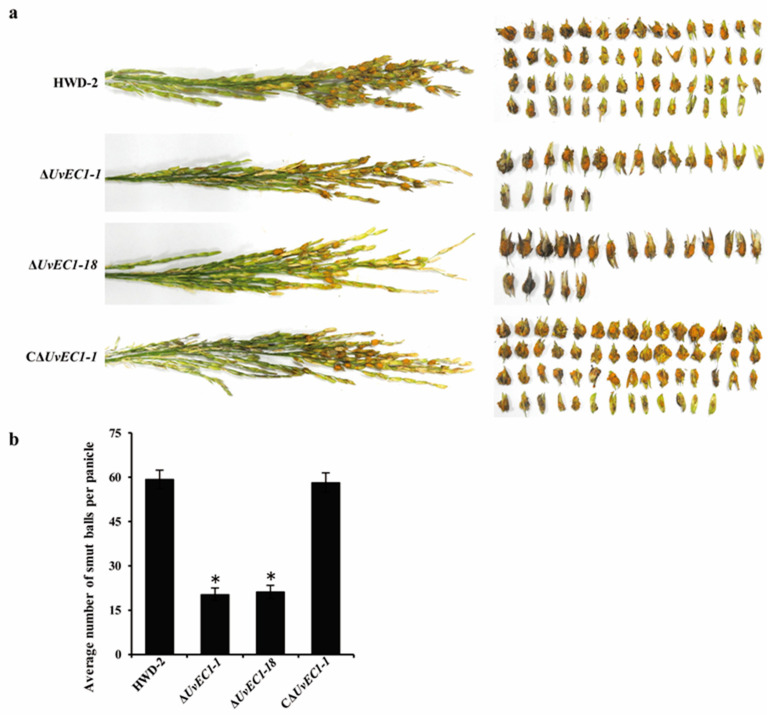
Deletion of *UvEC1* affects virulence of *U. virens*. (**a**) Virulence assays of HWD-2, the ∆*UvEC1* mutants and the complementation strain on rice spikelets (cv. Wanxian98) at 21 days post-inoculation (dpi). Left, entire panicles; right, individual spikelets from an infected panicle. (**b**) Average numbers of smut balls per panicle. Asterisks represent significant differences relative to HWD-2, as determined by LSD at *p* = 0.05.

To investigate the roles of *UvEC1* in the virulence of *U. virens*, we assessed the pathogenicity of all strains by inoculation on susceptible rice (cultivar ‘Wanxian98’) panicles at the early booting stage. At 21 days post inoculation (dpi), we observed the typical symptoms consisting of yellow smut balls on rice spikelets infected with HWD-2, ∆*UvEC1* mutants and complementation strain (Figure 5a). However, their number per panicle was much lower in plants infected with the ∆*UvEC1* mutants than in plants infected with either the WT or complementation strains (Figure 5b). Thus, deletion of *UvEC1* reduces the virulence of *U. virens*.

### 2.6. UvEC1 Is Highly Expressed during Infection by U. virens

**Figure 6:**
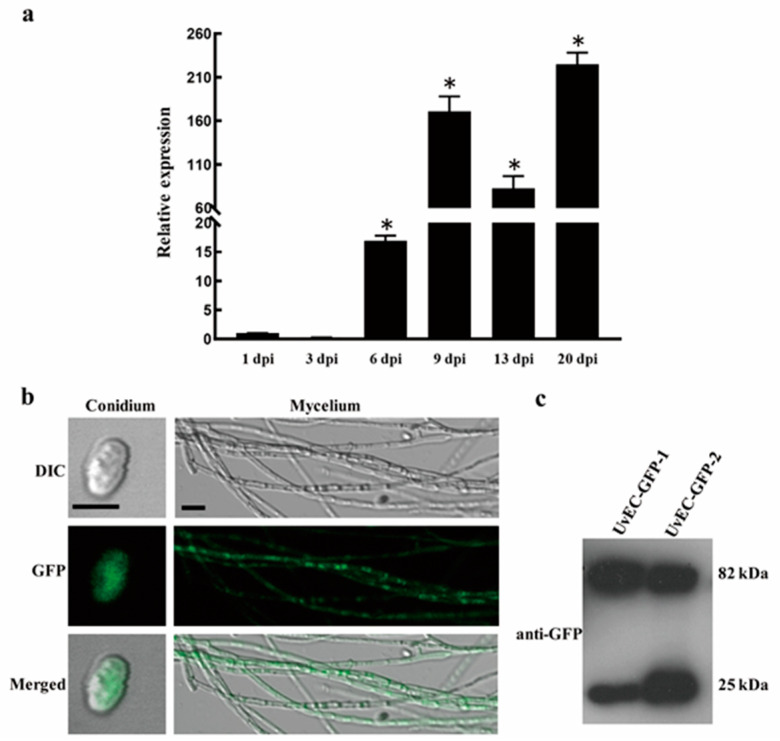
Expression of *UvEC1* and subcellular localization of UvEC1 in *U. virens.* (**a**) Relative expression of *UvEC1* at different infection stages on rice spikelets (1–21 d), as determined by RT-qPCR. *UvEC1* expression was normalized to that of *β-tubulin*. Asterisks represent significant differences relative to 1 dpi by LSD at *p* = 0.05 (**b**) Subcellular localization of UvEC1 in *U. virens*. DIC, differential interference contrast; GFP, green fluorescent protein. Scale bars = 10 μm. (**c**) Immunoblot analysis of UvEC1-GFP-CDC11 in the C∆*UvEC1-1* strain.

We next examined the relative expression of *UvEC1* over the course of the infection process by *U. virens* using RT-qPCR, using samples from infected spikelets collected at 1, 3, 6, 9, 13 and 21 dpi (Figure 6a). We found that *UvEC1* is highly expressed at 9 dpi and 20 dpi, with a 2- to 3-fold drop at 13 dpi. These results suggest that *UvEC1* is differently expressed during the infection process and is likely important for the pathogenicity of *U. virens*.

### 2.7. Subcellular Localization of UvEC1

To define the subcellular localization of UvEC1 in *U. virens*, we subjected the complementation strain C∆*UvEC1-1* to laser scanning confocal microscopy (LSCM), which revealed strong GFP signal in the cytoplasm of hyphae and conidia (Figure 6b). Immunoblot analysis of C∆*UvEC1-1* using an anti-GFP antibody detected a 66-kDa band corresponding to UvEC1-GFP, in addition to a band consistent with free GFP. This result indicates that the UvEC1-GFP fusion protein is partially cleaved in this strain (Figure 6c) and that UvEC1 accumulates in the cytoplasm.

### 2.8. Functional Enrichment Analysis of the Differentially Accumulating Proteome of the ∆UvEC1-1 Mutant Relative to HWD-2

**Figure 7:**
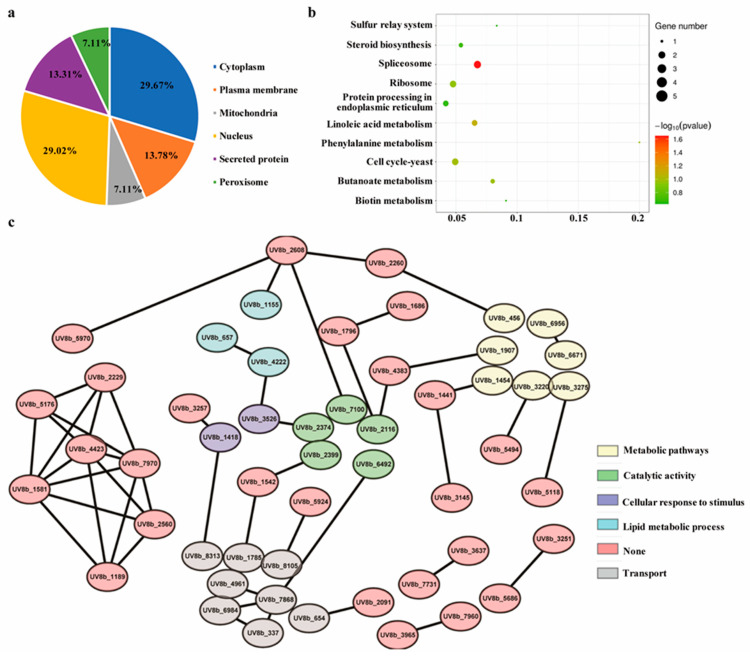
Prediction of subcellular localization, protein–protein interaction (PPI) map and functional annotation of differentially accumulating proteins (DAPs). (**a**) Predicted subcellular localizations of DAPs. (**b**) KEGG pathway analysis of DAPs. (**c**) Protein–protein interaction (PPI) network of DAPs.

We performed a quantitative proteomics analysis to identify differentially accumulating proteins (DAPs) between HWD-2 and the ∆*UvEC1-1* mutant, resulting in a list of 3967 proteins across three independent biological replicates based on our selection criteria for identification (Table S2). We observed high correlations across all replicates for a given genotype, confirming the quality of our dataset (Figure S2a). We independently validated the quality of the dataset by calculating the mass errors for enriched peptides. Most mass errors measured were smaller than 10 ppm, indicating a high degree of accuracy in the mass spectrometry (MS) data (Figure S2b). The lengths of most identified peptides varied from 7 to 20 amino acids (Figure S2c). Based on tandem mass tag (TMT)-based quantitative proteomics, we considered proteins with a fold change over 1.2 and a *p*-value lower than 0.05 as DAPs, when comparing their abundance between the mutant and the wild-type strain HWD-2 (Table S3). Volcano plot and heatmap analysis showed that 145 and 88 proteins were more and less abundant, respectively, in the ∆*UvEC1-1* mutant (Figure S3a,b). We performed in silico subcellular compartment predictions and discovered that among the 233 DAPs, most localized to the cytoplasm (29.7%), nucleus (29.0%), mitochondria (7.1%) or plasma membrane (13.8%), were secreted proteins (13.3%) or localized to the peroxisome (7.1%) (Figure 7a). Gene ontology (GO) enrichment analysis indicated that under the category of biological processes, DAPs were mainly associated with metabolic process, single-organism process and localization (Figure S4). In terms of molecular functions, DAPs were mostly related to binding, catalytic activity and transporter activity (Figure S4). In agreement with our subcellular compartment predictions, DAPs were mainly enriched in cell, organelle part, membrane and extracellular region terms from GO cellular components (Figure S4). Notably, Kyoto Encyclopedia of Genes and Genomes (KEGG) pathway enrichment analysis of DAPs revealed the spliceosome as a significantly enriched functional category (Figure 7b). We generated a protein interaction network of all DAPs based on the STRING database. DAPs formed a highly interconnected protein network. Several complexes and cellular functions established prominent and highly connected clusters, including metabolic pathways, catalytic activity, cellular response to stimulus, lipid metabolic process and transport (Figure 7c).

### 2.9. UvEC1 Is Involved in the Spliceosome

**Figure 8:**
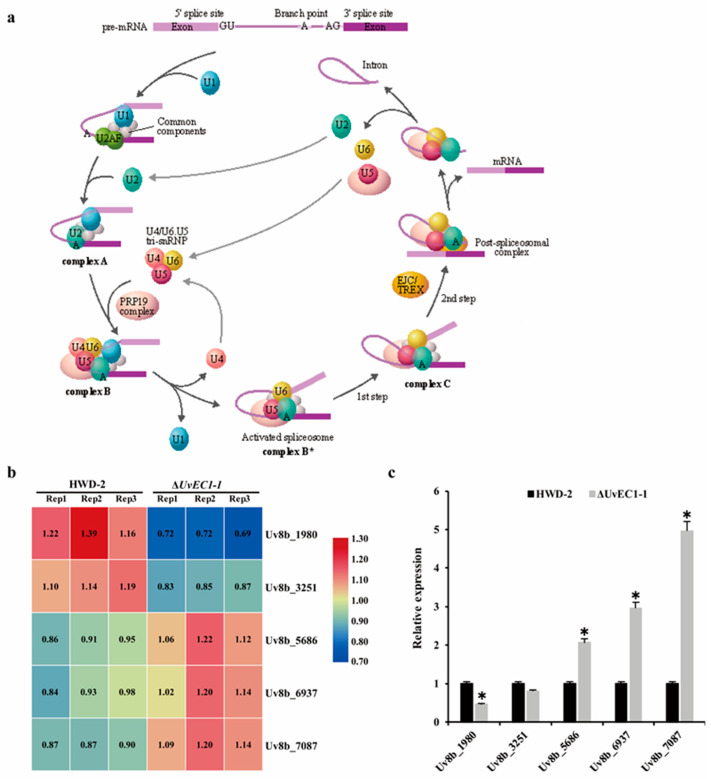
Deletion of *UvEC1* affects protein abundance and expression of spliceosome components. (**a**) Spliceosome pathway in *U. virens*. (**b**) Heatmap analysis of spliceosome protein levels in ∆*UvEC1-1* mutant and wild type HWD-2. (**c**) Relative expression of spliceosome genes in the ∆*UvEC1-1* mutant and wild type HWD-2 by RT-qPCR. Asterisks represent significant differences relative to HWD-2, as determined by LSD at *p* = 0.05.

Most eukaryotic genes are expressed as precursor mRNAs (pre-mRNAs) that are converted to mRNA via splicing, an essential step of gene expression during which noncoding sequences (introns) are removed and coding sequences (exons) are ligated together. Pre-mRNA splicing is catalyzed by the spliceosome, a multi-megadalton ribonucleoprotein (RNP) complex comprised of five small nuclear RNPs (snRNPs) and numerous proteins. The U2-dependent spliceosome is assembled from the U1, U2, U5 and U4/U6 snRNPs and multiple non-snRNP proteins (Figure 8a). During the splicing reaction, a variety of protein-nucleic acid complexes and splicing factors are combined and depolymerized in a highly precise sequence to form a pre-assembled complex U1, U2, U5 and U4/U6. Among our DAPs, we identified five proteins involved in the spliceosome: Uv8b_1980 (U5 snRNPs), Uv8b_3251 (U6 snRNPs), Uv8b_5686 (U1 snRNPs), Uv8b_6937 (U2 snRNPs) and Uv8b_7087 (U4 snRNPs) (Figure 8b). Based on RT-qPCR, these genes displayed altered relative transcript levels in the ∆*UvEC1-1* mutant compared to the wild type HWD-2 (Figure 8c). These results suggest that UvEC1 play a role in the spliceosome.

### 2.10. UvEC1 Is Associated with Toxin Production

**Figure 9:**
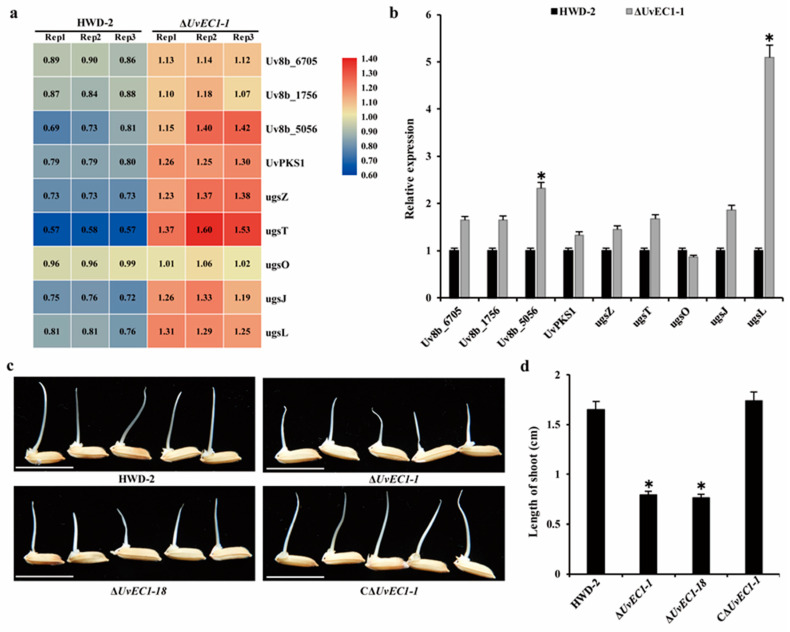
Deletion of *UvEC1* affects the protein accumulation and expression of toxin-encoding genes. (**a**) Heatmap analysis of levels of toxin-biosynthesis-related proteins in the ∆*UvEC1-1* mutant and HWD-2. (**b**) RT-qPCR analysis of toxin-biosynthesis-related genes in HWD-2 and ∆*UvEC1*. *β-tubulin* was used as reference gene. (**c**) Reduced inhibition of rice seed germination by culture filtrates of the ∆*UvEC1* mutants. Shoot lengths were measured after 5 d at 28 °C under illumination in growth incubators. (**d**) Mean length of rice shoots treated with filtrates from the indicated cultures. 50 rice seeds were shifted to 25-mL culture filtrates at 28 °C for 5 d. Three independent experiments (*n* = 100) were carried out (mean ± SD), bar =10 mm. Asterisks represent significant differences relative to HWD-2, as determined by LSD at *p* = 0.05.

Ustilaginoidins, which are toxic to plants, animals and humans, are one of the major types of mycotoxins produced by *U. virens*, e.g., ustilaginoidins O, E and F. These toxins are also toxic to rice seeds [29]. The *Ugs* gene cluster contains the polyketide synthase gene *UvPKS1* responsible for ustilaginoidin biosynthesis in *U. virens*. We noticed that several DAPs with higher abundance in the ∆*UvEC1-1* mutant are involved in toxin biosynthesis (Figure 9a). RT-qPCR analysis indicated that the genes encoding these proteins were more highly expressed in the ∆*UvEC1-1* mutant than in wild-type HWD-2 (Figure 9b). To assess the inhibitory effect of toxic compounds produced by *U. virens* on the germination of rice seeds, we isolated culture filtrates from PSB cultures of wild type HWD-2, the ∆*UvEC1-1* and ∆*UvEC1-18* mutants, and the complementation strain C∆*UvEC1-1*. We then treated rice seeds with these filtrates and measured shoot growth. The shoots of rice seedlings exposed to filtrates from the ∆*UvEC1* mutants were significantly shorter than those of seedlings treated with WT or the C∆*UvEC1-1* strain (Figure 9c,d). These findings suggest that the ∆*UvEC1* deletion mutants may have accumulated more toxic compounds, which limited shoot growth of rice seedlings during seed germination.

## 3. Discussion

Analyses of the *U. virens* genome and of the *U. virens* transcriptome upon infection of rice plants have illustrated the many virulence factors present in this fungus [11], only some of which have been experimentally verified. Characterizing additional genes participating in the pathogenic mechanism of *U. virens* will deepen our understanding and provide clues for the development of more effective strategies to control the disease. For example, the transcription factor UvPRO1 plays an important role in hyphal growth, conidiation, stress response and pathogenesis in *U. virens* [30], while the transcription factor UvHox2 regulates chlamydospore formation, conidiation and pathogenicity [31]. The transcription factor UvCom1 regulates the formation of smut balls on rice spikelets, and deletion of *UvCom1* also significantly affected vegetative growth, conidiation and stress response in *U. virens* [32]. Likewise, silencing of the low-affinity iron transporter Uvt3227 resulted in decreased sporulation and increased pathogenicity [33]. The Bax inhibitor UvBI-1 controls mycelial growth, conidiation, stress response and pathogenicity in *U. virens* [34]. UvAc1 and UvPdeH control intracellular cAMP levels, development and pathogenicity [35]. UvPmk1 and UvCDC2 play important roles in pathogenicity, mycelium growth, conidiation and stress response [36]. The cellular morphogenetic protein UvPal1 affects hyphal growth, cell morphology, stress adaptation and virulence, and UvPal1 physically interacts with the cell division control protein UvCdc11 (also called septin) to mediate the formation of the septin complex to maintain cellular morphology and virulence [12]. Autophagy 8 (UvAtg8)-mediated autophagy plays important roles in growth, stress responses, production of toxic compounds, conidiation, secondary spore formation and pathogenicity in *U. virens* [37]. The histone deacetylase UvRpd3 and the mitogen-activated protein kinase (MAPK) UvSlt2 affect *U. virens* virulence*,* and both proteins interact to remove 2-hydroxyisobutyrylation and acetylation marks from UvSlt2 [38].

In this study, we characterized the putative ester cyclase UvEC1. A BLASTP search for EC1 homologs revealed that EC1 is specifically distributed in bacteria and a few fungi, as we failed to identify proteins with homology to UvEC1 encoded by the genomes of others important plant pathogens, such as *Magnaporthe oryzae*, *Fusarium graminearum* and *Botrytis cinerea*. The deletion of *UvEC1* significantly affected conidiation, vegetative growth, pathogenicity and stress responses in *U. virens*. We also performed a TMT-based quantitative proteomics analysis to identify DAPs between the Δ*UvEC1-1* mutant and the wild-type strain HWD-2. Bioinformatics analyses revealed that several DAPs are involved in metabolism, protein localization, catalytic activity, binding and the spliceosome. Many DAPs are secreted proteins (Figure S5), we speculate that UvEC1 may contribute to the secretion of secreted proteins, which may themselves play important roles in the pathogenic process of *U. virens*. These secreted proteins should be investigated in future for their role in pathogenicity.

Genetically encoded resistance is considered to be the most efficient approach in controlling RFS. As no single resistance gene or fully immune rice varieties against RFS have been identified, rice false smut disease is difficult to control. Recently, an RNA interference (RNAi)-based approach termed host-induced gene silencing (HIGS) has been developed to generate resistant cultivars as a means to control fungal diseases, whereby small interfering RNAs (siRNAs) that match important genes from the invading pathogen are produced by transgenic host plants to silence fungal genes during infection [39]. HIGS has increasingly become one of the most practical technologies to generate new disease-resistant crop varieties due to its high specificity and efficiency in silencing pathogen-derived genes [40,41]. For example, HIGS directed at the virulence genes *SGE1*, *FGP1* and *STE12* from *F. graminearum* conferred resistance to *Fusarium* head blight in wheat (*Triticum aestivum*) [40]. Likewise, PsFUZ7, which encodes MAPK kinase, is an important pathogenicity factor that regulates infection and development of *Puccinia striiformis* f. sp. *Tritici* (*Pst*), the agent causing wheat stripe rust; HIGS of *PsFUZ7* confers stable resistance to wheat stripe rust [42]. Barley stripe mosaic virus (BSMV)-mediated HIGS silencing of three predicted pathogenicity genes, encoding a MAPK, a cyclophilin and a calcineurin regulatory subunit, suppressed the disease phenotypes associated with wheat leaf rust [43]. Similarly, HIGS of *CPK1,* corresponding to *Pst_13661* in the *Pst* genome, enhanced what resistance to stripe rust [44,45]. Clearly, a key step in developing a successful HIGS strategy is to identify effective target genes in phytopathogens. We believe that *UvEC1* might thus be a suitable target to reduce or control rice false smut development in rice via HIGS. 

In summary, we found that deletion of *UvEC1* affects the development and virulence of *U. virens*. Ester cyclase is a type of polyketide synthases, and polyketide synthases has been reported in *U. virens* to participate in the biosynthesis of ustilaginoidin, our results showed that UvEC1 negatively regulate ustilaginoidin-related synthases. Quantitative proteomics data show that UvEC1 has multiple effects on metabolism, protein localization, catalytic activity, binding, toxin biosynthesis, and spliceosome.

## 4. Materials and Methods

### 4.1. Fungal Strains and Growth Conditions

The wild-type strain HWD-2, which is fully virulent on the rice cultivar ‘Wanxian-98’, was used in this study [46]. All transformed strains derived from HWD-2 were cultured on potato sucrose agar (PSA) at 28 °C in the dark. Seven-day-old mycelia were collected from potato sucrose broth (PSB) cultures with shaking at 180 rpm and used for isolation of fungal DNA/RNA. Conidia were separated for Agrobacterium (*Agrobacterium tumefaciens*)-mediated transformation [30].

### 4.2. Gene Deletion and Complementation in U. virens

To generate *UvEC1* gene deletion mutants, approximately 1.5 kb of flanking sequences upstream and downstream of *UvEC1* were ligated into the pGKO vector to generate the final deletion vector pGKO-UvEC1. The construct was introduced into Agrobacterium strain EHA105. The resulting Agrobacteria were then used to transform WT conidia. Hygromycin-resistant transformants were isolated and screened by PCR with primers P1/P2 and then confirmed by RT-PCR and Southern blot analysis. For complementation assays, an approximately 2.5-kb fragment containing a 2-kb native promoter region and the full-length *UvEC1* coding region was cloned into vector p3300neo-GFP. Agrobacterium strain EHA105 harboring the resulting p3300neo-UvEC1-GFP vector was used for Agrobacterium-mediated transformation by co-cultivation with conidia from the Δ*UvEC1-1* mutant. Transformants were selected on PSA medium with 500 μg/mL G418. Complementation strains were screened by conventional PCR with primers P1/P2 and then confirmed by immunoblotting. The subcellular localization of UvEC1 was determined by observing the GFP signal in the mycelium of transformants on a Zeiss LSM 510 Meta confocal microscope (Carl Zeiss). All primers used in this study are listed in Table S1.

### 4.3. DNA/RNA Manipulation, Southern Blotting and RT-qPCR

Genomic DNA from mycelium was extracted by the CTAB method [47]. Southern blotting was performed according to the protocol provided with the Amersham Gene Images with Alkphos Direct Labelling and Detection System (GE Healthcare Life Science, Marlborough, MA, USA). Total RNA was extracted with TRIzol reagent (Vazyme Biotech, Nanjing, China) from mycelia collected from cultures grown for 7 days on PSB, or from infected rice spikelets on different days (1, 3, 6, 9, 13 or 21 dpi). First-strand cDNAs were synthesized using a TransScript ^®^ One-Step gDNA Removal and cDNA Synthesis SuperMix (TransGen Biotech, Beijing, China). RT-qPCR was conducted using the TransStart^®^ Tip Green qPCR SuperMix (TransGen Biotech, Beijing, China). Primer pair P5/P6 was designed to measure *UvEC1* expression levels (Table S1). All results were normalized to those for *β-tubulin* (UV8b_900, GenBank ID KDB18052), and relative changes in gene expression levels were calculated by the comparative Ct method (Applied Biosystems, Foster City, CA, USA).

### 4.4. Vegetative Growth, Conidiation and Pathogenicity

For vegetative growth, 5-mm (diameter) mycelial plugs were transferred from 14-day-old PSA plates and grown on fresh PSA medium at 28 °C. After 14 d of incubation, the radial growth of vegetative mycelia was measured. For conidial production, strains were grown in PSB medium at 28 °C. After shaking at 180 rpm for seven days, the cultures were filtered through four layers of gauze, and conidial production was measured using a hemocytometer. The virulence of *U. virens* strains was tested based on the inoculation method of Lv et al. [24]. The rice cultivar Wanxian-98 was inoculated with 2 mL of mycelial and spore suspension using a syringe in the middle section of distal internodes at the 8th stage of panicle development. Inoculated rice plants were placed in a greenhouse with a relative humidity of 95 ± 5% and temperature of 28 ± 2 °C. Each treatment was repeated three times.

### 4.5. Stress Adaptation Assays

To test adaptation to stress, 5-mm (diameter) mycelial plug from PSA plates were placed on fresh PSA plates (control) or PSA plates containing 0.3 M NaCl, 0.5 M sorbitol, 120 µg/mL calcofluor white (CFW), 120 µg/mL Congo red (CR), 0.03% SDS (*w/v*), 0.02% H_2_O_2_ or 0.04% H_2_O_2_. Plates were incubated in the dark for 14 days to observe colony morphology and measure mycelium growth rates. The experiment was repeated three times in each group.

### 4.6. Immunoblotting

Approximately 200 mg of finely ground mycelia was resuspended in 1 mL of lysis buffer (10 mM Tris-HCl [pH 7.5], 1 mM EDTA, 1 mM phenylmethylsulfonyl fluoride [PMSF]) and 10 μL of protease inhibitor cocktail (Sangon, Shanghai, China). Proteins were separated by SDS-PAGE and transferred onto a polyvinylidene fluoride membrane with a Bio-Rad electroblotting apparatus. Immunoblot analysis was performed with an anti-GFP primary antibody (1:5,000, Sigma-Aldrich, United States) and an anti-rabbit horseradish peroxidase-conjugated secondary antibody (1:10,000, Sigma-Aldrich, United States). Luminescence signals were detected using Pierce ECL western blotting substrate (Thermo Fisher Scientific, Hillsboro, OR, USA) with a ChemiDoc XRS + system (Bio-Rad Laboratories, Hercules, CA, USA).

### 4.7. Protein Extraction and Digestion

Proteins were extracted in lysis buffer (1% Triton X-100, 10 mM dithiothreitol, 1% Protease Inhibitor Cocktail, 50 μM PR-619 [inhibitor of deubiquitylating enzymes] and 2 mM EDTA). Proteins were dissolved in 8 M urea, and the protein concentration was determined with BCA kit (Beyotime Biotechnology, Shanghai, China) according to the manufacturer’s instructions. For digestion, the protein solution was reduced with 5 mM dithiothreitol for 30 min at 56 °C and alkylated with 11 mM iodoacetamide for 15 min at room temperature in the dark. The protein sample was then diluted by adding 100 mM triethylammonium bicarbonate (TEAB) to obtain a final urea concentration below 2 M. Finally, trypsin was added at a 1:50 trypsin-to-protein mass ratio for the first digestion overnight and a 1:100 trypsin-to-protein mass ratio for a second 4 h digestion. After trypsin digestion, peptides were desalted using a Strata X C18 SPE column (Phenomenex, Torrance, CA, USA) and vacuum-dried. Peptides were reconstituted in 0.5 M TEAB and processed according to the manufacturer’s protocol for the TMT kit (Thermo Fisher Scientific, Hillsboro, OR, USA).

### 4.8. LC-MS/MS Analysis

Peptides were first dissolved in solvent A (0.1% formic acid [FA], 2% acetonitrile [can], 98% H_2_O). The peptide solution was then loaded onto a reversed-phase analytical column (homemade, 100 μm ID × 15 mm long, 2 μm particle, 100 Å). Peptides were separated with a gradient of 6% to 22% solvent B (0.1% FA in 98% ACN) for 24 min, 22% to 36% for 10 min and climbing to 80% in 3 min then holding at 80% for the last 3 min. The flow rate was set to 400 nL/min on an EASY-nLC 1000 UPLC system (Thermo Fisher Scientific, USA). Peptides were subjected to nanospray ion source followed by tandem mass spectrometry (MS/MS) in Q ExactiveTM Plus (Thermo Fisher Scientific, USA) coupled online to the UPLC. The applied electrospray voltage was 2.0 kV. The m/z scan range was 350 to 1800 for full scans, and intact peptides were detected in an Orbitrap mass analyzer at a resolution of 70,000. Peptides were then selected for MS/MS using normalized collision energy (NCE) setting of 28 and the fragments were detected in the Orbitrap at a resolution of 17,500. A data-dependent procedure that alternated between one MS scan followed by 20 MS/MS scans was applied to the top 20 precursor ions (Threshold ion count: 1.5 × 10^4^; dynamic exclusion: 15 s; electrospray voltage: 2.0 kV; Automatic gain control: 5 × 10^4^ ions; m/z scan range: 350 to 1600.) The resulting MS/MS data were searched using MaxQuant software (version 1.5.2.8, http://www.maxquant.org/, accessed on 14 April 2021) against the UniProt_*U. virens* protein database (www.ncbi.nlm.nih.gov/nuccore/JHTR00000000, accessed on 14 April 2021). Trypsin/P was specified as the cleavage enzyme, allowing up to four missing cleavages. The mass tolerance for precursor ions was set to 30 ppm in the first search and 10 ppm in the main search, and the mass tolerance for fragment ions was set to 0.02 Da. False discovery rate thresholds for peptides were less than 1%, minimal peptide length below seven amino acids, peptides with a score below 40.

### 4.9. Bioinformatics Analysis

The *UvEC1* sequence was downloaded from the *U. virens* database (https://www.ncbi.nlm.nih.gov/nuccore/JHTR00000000, accessed on 14 April 2021). Conserved protein domains were predicted with the online SMART tool (http://smart.embl-heidelberg.de/, accessed on 14 April 2021). Other protein sequences from different organisms were obtained from the National Center for Biotechnology Information (NCBI). After sequence alignment, a phylogenetic tree was generated with Mega 7.0 Beta using the neighbor-joining algorithm. Dendrograms were generated, and the percentage of replicate trees in which the associated sequences clustered together by bootstrap analysis (1000 replicates) is shown on the corresponding branches. Bioinformatics analyses of Gene Ontology (GO) annotation of the proteome was achieved with the UniProt-GOA database (http://www.ebi.ac.uk/GOA/, accessed on 14 April 2021). Proteins were classified by GO annotation based on three categories: biological process, cellular component and molecular function. For each category, a two-tailed Fisher’s exact test was used to test enrichment of differentially modified proteins against all identified proteins. Each GO term with a corrected *p*-value < 0.05 was considered significant. The Kyoto Encyclopedia of Genes and Genomes (KEGG) database was used to identify enriched pathways by a two-tailed Fisher’s exact test among differentially modified proteins against all identified proteins. Each pathway with a corrected *p*-value < 0.05 was considered significant. These pathways were classified into hierarchical categories according to the KEGG website. WoLF PSORT (version 0.2, http://www.genscript.com/psort/wolf_psort.html, accessed on 14 April 2021) was used to predict protein subcellular localization. Each differentially accumulated protein was queried against the STRING database (version 10.5, https://string-db.org/, accessed on 14 April 2021) for protein–protein interactions. Only interactions whereby both proteins belonged to our dataset were selected, thus excluding external candidates. STRING defines a metric called “confidence score” to define the confidence of interaction; all interactions with a confidence score > 0.7 (high confidence) were retained. The interaction network from the STRING database was visualized in R with the package networkD3 (version 0.4, https://cran.r-project.org/web/packages/networkD3/, accessed on 14 April 2021).

### 4.10. Statistical Analysis

Data were subjected to analyses of variance (ANOVA) using SPSS 14.0 software (SPSS, Chicago, IL, USA). For statistically significant effects, means were separated using the least significant difference (LSD) test (*p* = 0.05).

## Data Availability

The mass spectrometry proteomics data have been deposited to the ProteomeXchange Consortium via the PRIDE partner repository with the dataset identifier PXD024701.

## Supplementary Materials

The following are available online at https://www.mdpi.com/article/10.3390/ijms22084069/s1.
