# Supplementary material for: Quantitative Proteomics Analysis Reveals the Function of the Putative Ester Cyclase UvEC1 in the Pathogenicity of the Rice False Smut Fungus Ustilaginoidea virens
Source: Int J Mol Sci. 2021 Apr 15;22(8):4069. doi: 10.3390/ijms22084069 (PMC8071170; doi:10.3390/ijms22084069)
Supplement: Supplementary file 1 [file ijms-22-04069-s001.zip › ijms-1183058 -supplementary figures.pdf]

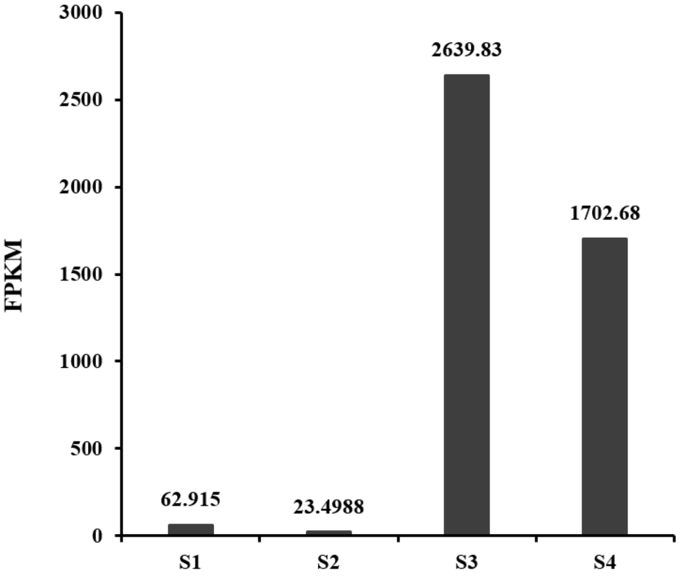

Figure S1. The expression levels of *UvEC1* at four different infection stages from RNA-seq data. FPKM, Fragments Per Kilobase per Million. S1, 1 dpi; S2, 3 dpi; S3, 6 dpi; S4, 15 dpi.

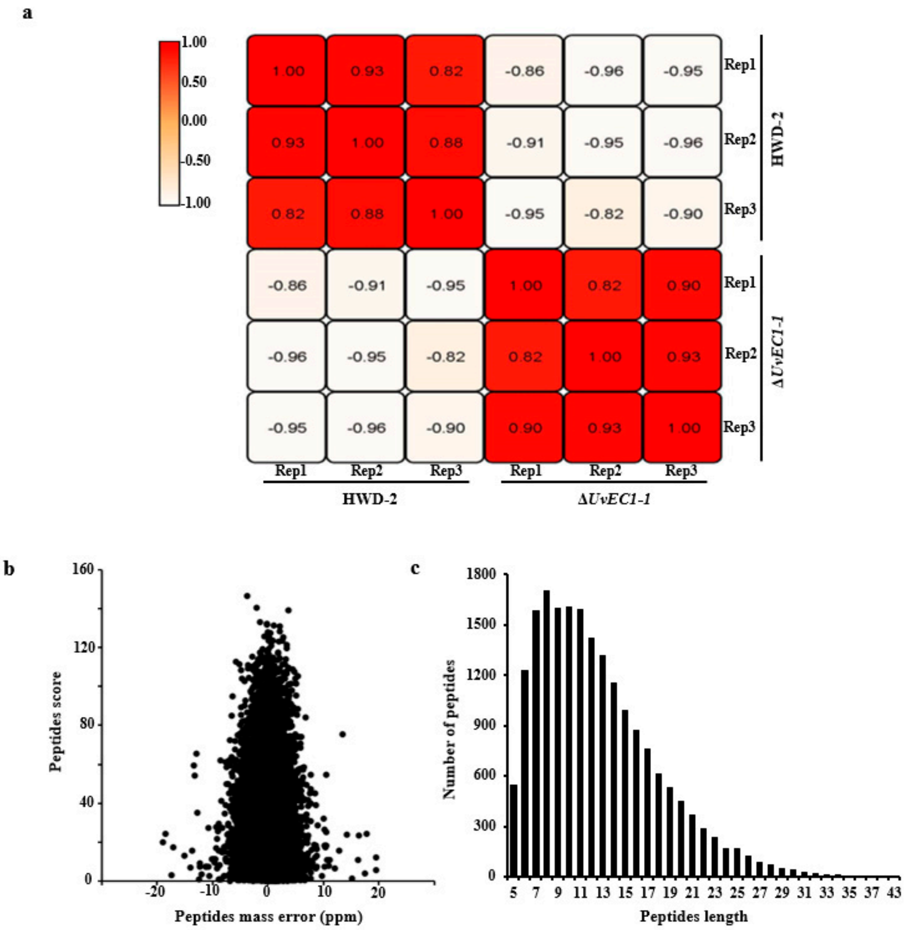

Figure S2. Reproducibility of quantitative proteome analyses. (a) Pearson's correlation coefficients (*r*) between individual biological replicates of the proteomics data between all six samples. (b) Distribution of mass error of peptides from identified DAPs. (c) Distribution of peptide lengths for DAPs from the MS data.

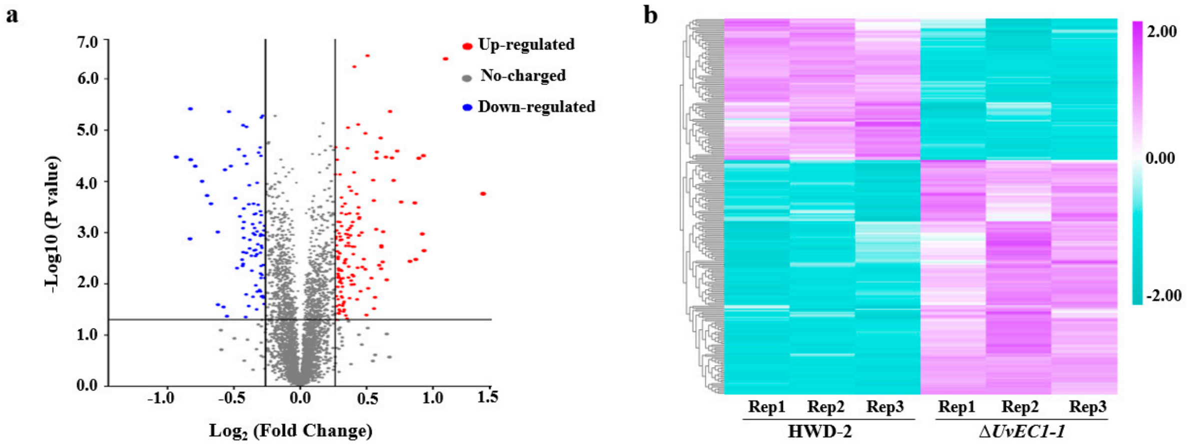

Figure S3. Changes in the proteome between HWD-2 and the Δ*UvEC1-1* mutant. (a) Volcano plot of proteins with significantly higher (fold change > 1.2, $p < 0.05$) or lower (fold change > 1.2, $p < 0.05$) abundance in the Δ*UvEC1-1* mutant relative to HWD-2. (b) Heatmap analysis of protein abundance in the Δ*UvEC1-1* mutant and wild type HWD-2.

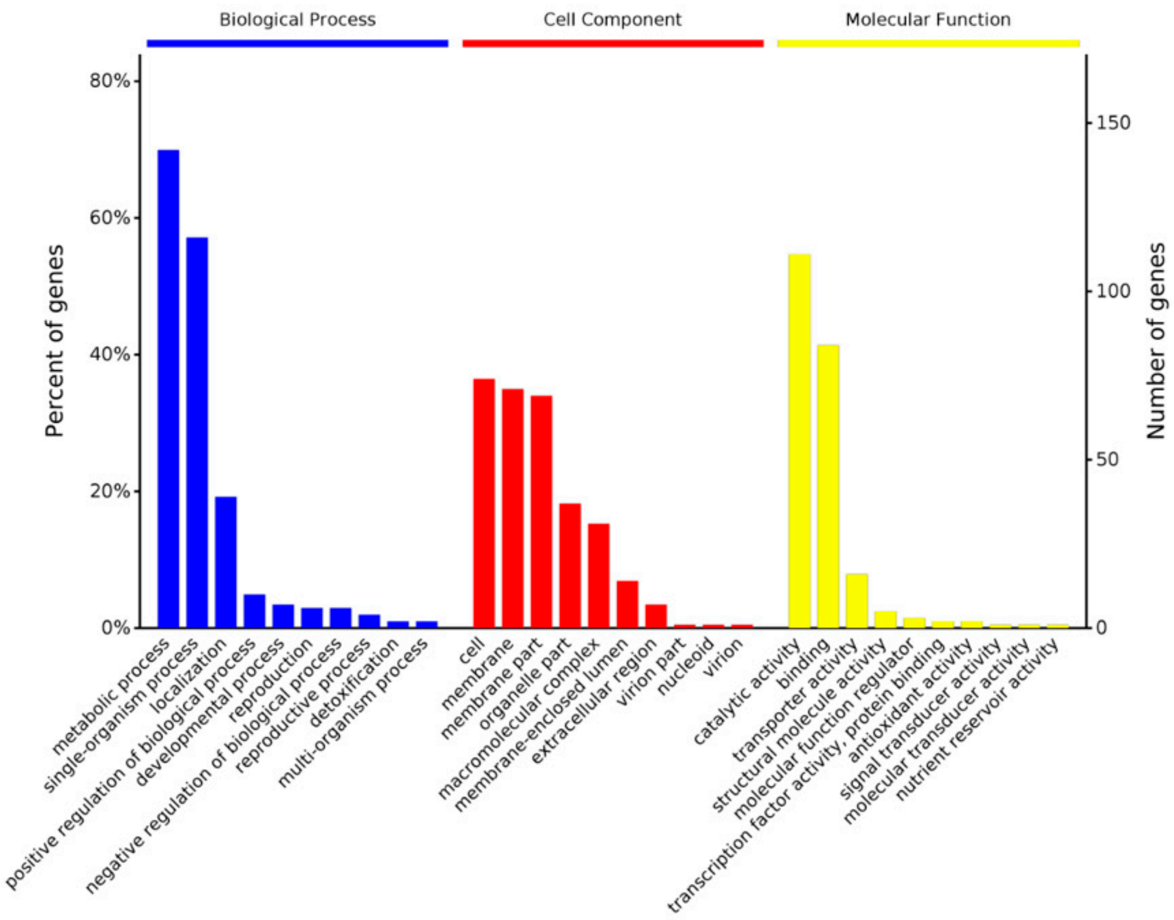

Figure S4. GO enrichment analysis of DAPs for biological processes, molecular functions, and cellular components.

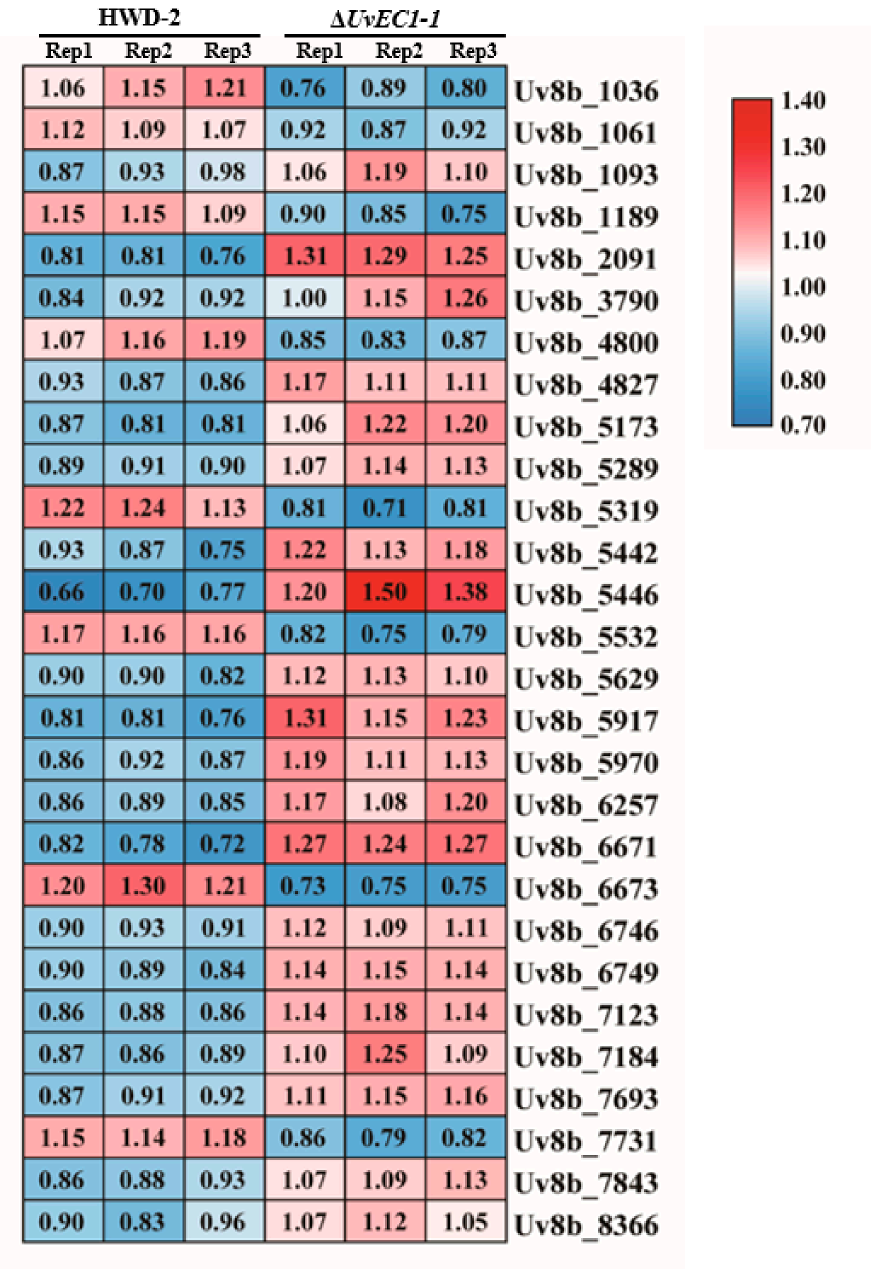

Figure S5. Heatmap analysis of levels of secreted proteins in the Δ*UvEC1-1* mutant and HWD-2.
